# Blockade of Rho-associated protein kinase (ROCK) inhibits the contractility and invasion potential of cancer stem like cells

**DOI:** 10.18632/oncotarget.15248

**Published:** 2017-02-10

**Authors:** Srisathya Srinivasan, Vandhana Ashok, Sagarajit Mohanty, Alakesh Das, Sreya Das, Sushant Kumar, Shamik Sen, Rahul Purwar

**Affiliations:** ^1^ Department of Biosciences & Bioengineering, Indian Institute of Technology Bombay (IIT Bombay), Mumbai, Maharashtra, India

**Keywords:** ROCK pathway, cancer stem cells, cell contractility, invasion, metastasis

## Abstract

Recent studies have implicated the roles of cancer stem like cells (CSCs) in cancer metastasis. However, very limited knowledge exists at the molecular and cellular level to target CSCs for prevention of cancer metastasis. In this study, we examined the roles of contractile dynamics of CSCs in cell invasion and delineated the underlying molecular mechanisms of their distinct cell invasion potential. Using de-adhesion assay and atomic force microscopy, we show that CSCs derived from melanoma and breast cancer cell lines exhibit increased contractility compared to non-CSCs across all tumor types. In addition, CSCs possess increased ECM remodeling capacity as quantified by collagen degradation assay. More importantly, pharmacological blockade of Rho-associated protein kinase completely abolished the contractility and collagen degradation capacity of both CSCs and non-CSCs. In conclusion, our study demonstrates the importance of cell contractility in regulating invasiveness of CSCs and suggests that pharmacological targeting of ROCK pathway represents a novel strategy for targeting both CSCs and bulk population for the treatment of cancer metastasis.

## INTRODUCTION

Although surgical removal of primary tumors is effective in treating localized tumors, treating cancer metastasis remains a formidable challenge. Recent studies have described the roles of cancer stem cells (CSCs) in cancer metastasis [1–5]. Therefore, it is critical to target CSCs and tumor bulk population for long-term cancer remission. However, there exists limited knowledge of therapeutic targets, which can be used for targeting CSCs along with bulk population for the treatment of cancer metastasis.

Cancer metastasis is a multi-step process and involves several biochemical and biophysical cues of the tumor cells. Contractile dynamics of a tumor cell represents one of the most important biophysical properties that regulates metastasis and invasion ability [6], and is closely associated with cell spreading and cell adhesion properties [7]. Increased cell contractility in breast cancer is associated with cell invasion through dense interstitial matrices by allowing cells to squeeze through matrix pores and also in intravasation and extravasation [8]. We, and others have previously demonstrated a close relationship between cell contractility and invasiveness in breast cancer cell line (MDA-MB-231), ovarian cancer cell lines including OVCAR3 and SKOV3 and melanoma cells [9–12]. Additionally, pancreatic cell lines with higher contractility secrete increased levels of matrix metalloproteases (MMPs) and show higher invasion capacity [13]. However, it remains unknown if biophysical characteristics, especially contractile dynamics of CSCs are distinct compared to the bulk tumor population and contribute in CSC mediated cell invasion.

Cell contractility is mediated mainly by the acto-myosin machinery, and is generated by myosin II sliding along actin filaments [14]. Several studies have demonstrated that tumor cells having defective myosin bundles are deformed easier than normal cells [15, 16]. Myosin II activity is regulated by two groups of enzymes including myosin light chain kinase (MLCK) and Rho-associated protein kinase (ROCK), both of which phosphorylate myosin light chain (MLC). In addition, ROCK inhibits phosphatase activity [17].

Though contractile dynamics of tumor cells is regulated by ROCK pathway, its role in contractility and cell invasion potential of CSCs in various cancer has not yet been examined. In this study, we examined the invasion potential of CSCs, focusing on their contractile dynamics and ECM remodeling, to identify the novel pathway for targeting CSCs. We demonstrate higher contractile dynamics of CSCs compared with non-CSCs using trypsin de-adhesion assay, which we established earlier for probing contractile mechanics of adherent cells [7], and by atomic force microscopy (AFM). Interestingly, CSCs showed increased collagen degradation potential compared to the control population (parental cells). Importantly, pharmacological blockade of ROCK pathway led to inhibition of contractility and collagen degradation ability of both CSCs and non-CSCs across cancer types. In conclusion, pharmacological targeting of ROCK abrogates the contractility and cell invasion potential of both CSCs and non-CSCs, and is therefore a novel strategy for the treatment of cancer metastasis.

## RESULTS

### CSCs show higher contractile dynamics compared to parental cells

We enriched CSCs from two different breast cancer cell lines (MCF-7 and MDA-MB-231) and a melanoma cell line (MDA-MB-453) using two distinct approaches: Fluorescence activated cell sorting (FACS) and spheroid assay as described in materials and methods.

**Figure 1:**
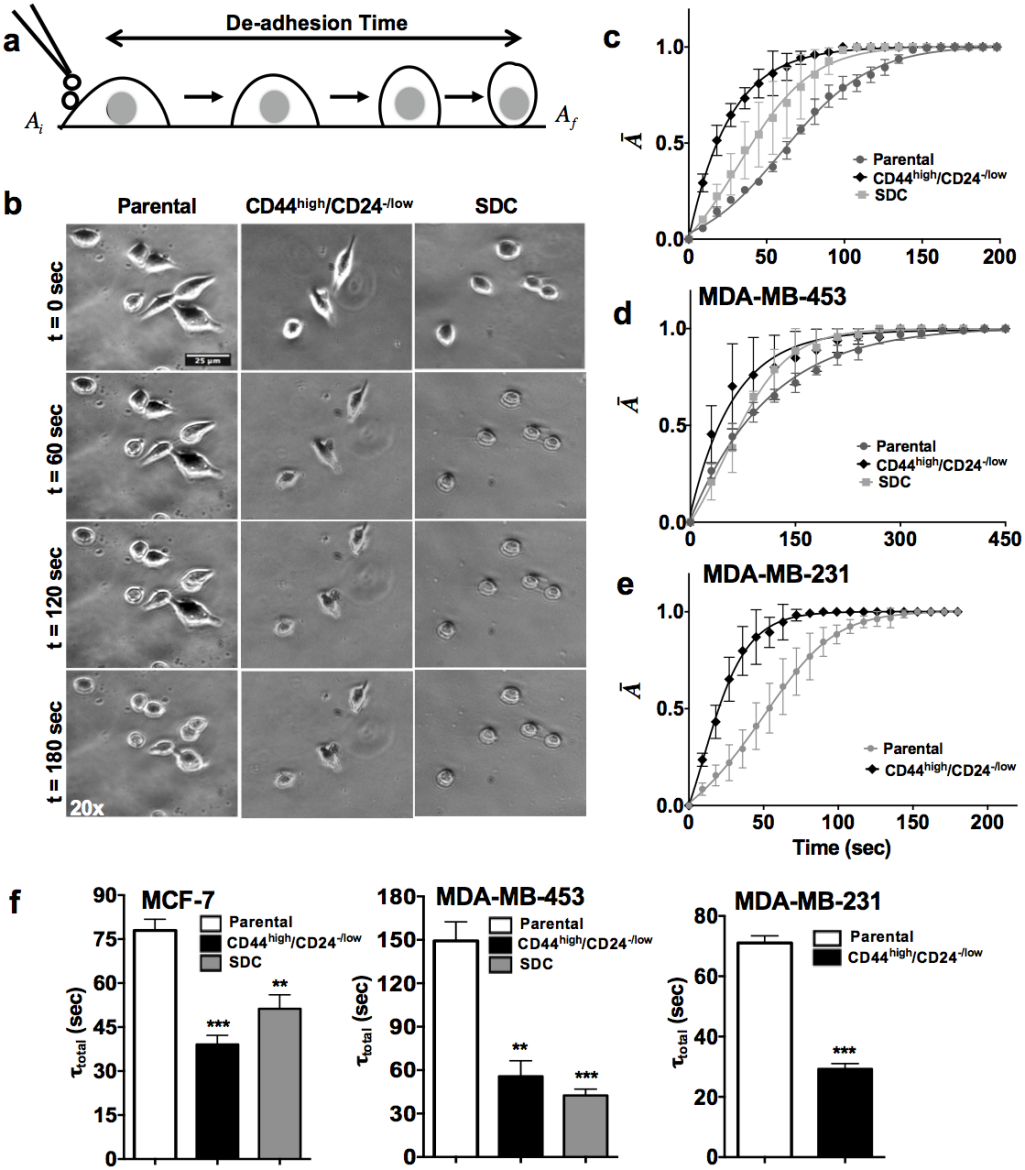
CSCs are more contractile than parental cells **a**. Schematic of the trypsin de-adhesion assay. Upon incubation with warm trypsin, adherent cells round up. The de-adhesion process is imaged till cells are completely rounded up but remain attached to their substrates. **b**. Representative time-lapse image of the de-adhesion dynamics of MCF-7 (parental cells, CD44^high/^CD24^−/low^ and SDC cells). Scale bars: 25 microns. **c-e**. De-adhesion data was quantified by plotting the normalized change in cell area (*Ā*) as a function of time for MCF-7, MDA-MB-453 and MDA-MB-231 CSCs and parental cells respectively. The detachment response was sigmoidal with CSCs like population enriched from all three cell lines showing higher contractility when compared to the parental cells. **f**. The detachment response curve was fit using the Boltzmann equation to obtain the time constants τ_1_ and τ_2_. The total de-adhesion time (τ_total_) was calculated as the sum of τ_1_ and τ_2_.. Faster de-adhesion dynamics resulted in a significant reduction in the τ_total_ for stem-like cells compared to the parental population. (**p < 0.001, *** p< 0.0001) (n = 3). Data is represented as Mean ± SEM for line graph and Mean + SEM for bar graph.

Because CD44^high^/CD24^−/low^ cells do not represent pan-CSC population, spheroid assay was also used to enrich CSCs [18]. To confirm that the spheroids were enriched with stem like cells, the mRNA levels of stemness genes (Oct4, Nanog and Klf4) were quantified by real time RT-PCR. The spheroids isolated from MCF-7 and MDA-MB-453 showed increase in the expression level of Oct4 (5-120 fold), Nanog (4 to 140 fold) and Klf4 (3.5 to 8 fold) (Supplementry Figure 1a).

As contractile dynamics is one of the most critical biophysical properties for the cell invasion potential, CSCs contractility was assessed using the trypsin de-adhesion assay, in which retraction kinetics of individual cells treated with warm trypsin is tracked till cells round up but remain attached to the underlying substrate (Figure 1a) [7]. Both, the CD44^high^/CD24^−/low^ cells and the spheroid derived single cells (SDCs) de-adhere faster when compared to the cells from the bulk population, as evident from the representative time-lapse images of MCF-7 (Figure 1b). For quantification of contractile dynamics of CSCs from all cell lines, temporal curves of normalized change in area (A¯) were fitted with the Boltzmann equation to obtain the de-adhesion time constants τ_1_ and τ_2_, respectively (Figure 1c-1e). The total de-adhesion time (τ_total_) was chosen as the sum of the two time constants. In line with the visual observations, statistically significant reduction was observed in the τ total (τ_1_+ τ_2_) between parental cells and stem like cells across all cell lines (Figure 1f). Specifically, de-adhesion time (τ total) for CD44^high^/CD24^−/low^ and the SDCs of MCF-7 cells was 40 sec and 50 sec respectively compared to 80 sec for parental cells (p value < 0.0001). In MDA-MB-453 and MDA-MB-231 cells, almost 50-60% drop in total de-adhesion time was observed in both CD44^high^/CD24^−/low^ cells and the SDCs compared to parental cells (p value < 0.0001). Collectively, these results demonstrate that CSCs are more contractile compared to the parental population across all cancer cell lines tested.

### Contractile dynamics of CSCs is regulated by the ROCK pathway

**Figure 2:**
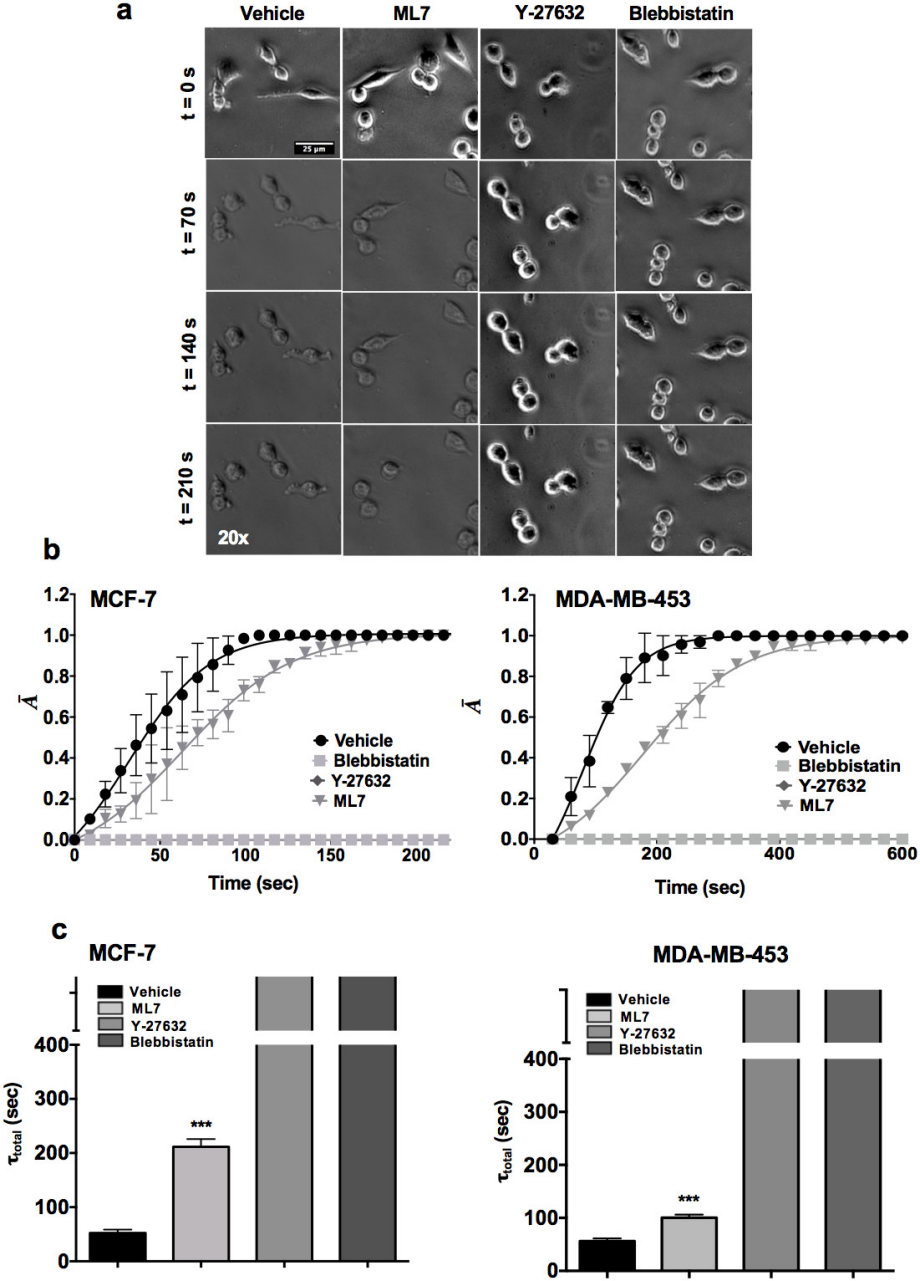
The ROCK pathway plays a dominant role in modulating CSC contractility **a**. Representative time-lapse image of spheroid derived single cells (SDC) of MCF-7 cell line treated with contractility inhibiting drugs ML7, Y-27632 and Blebbistatin (Scale bars: 25 microns). **b**. De-adhesion data of SDCs of MCF-7 and MDA-MB-453 cells treated with Y-27632 and Blebbistatin. **c**. Quantification of τ_total_ in control (vehicle) and drug-treated SDCs from MCF-7 and MDA-MB-453 respectively. (***, p value < 0.0001) (n = 20 cells per condition). Data is represented as Mean ± SEM for line graph and Mean + SEM for bar graph.

Next, we attempted to delineate the mechanisms of increased contractility of CSCs compared with parental cells. Given the higher contractility of stem-like cells (CD44^high^/CD24^−/low^ sorted cells and SDCs), we probed the relative contributions of MLCK and ROCK pathways in modulating contractile mechanics using highly specific pharmacological inhibitors (ML7, Y-27632 and Blebbistatin) of MLCK and ROCK pathway (Figure 2a). ML7 (MLCK inhibitor) delayed the de-adhesion dynamics of MCF-7-SDCs compared to vehicle treated controls (Figure 2b). However, treatment with either Y-27632 (ROCK inhibitor) or Blebbistatin (global myosin inhibitor) completely abolished de-adhesion of CSCs of MCF-7 and MDA-MB-453 cells with no observable changes in cell area even after 300 sec (Figure 2c).

**Figure 3:**
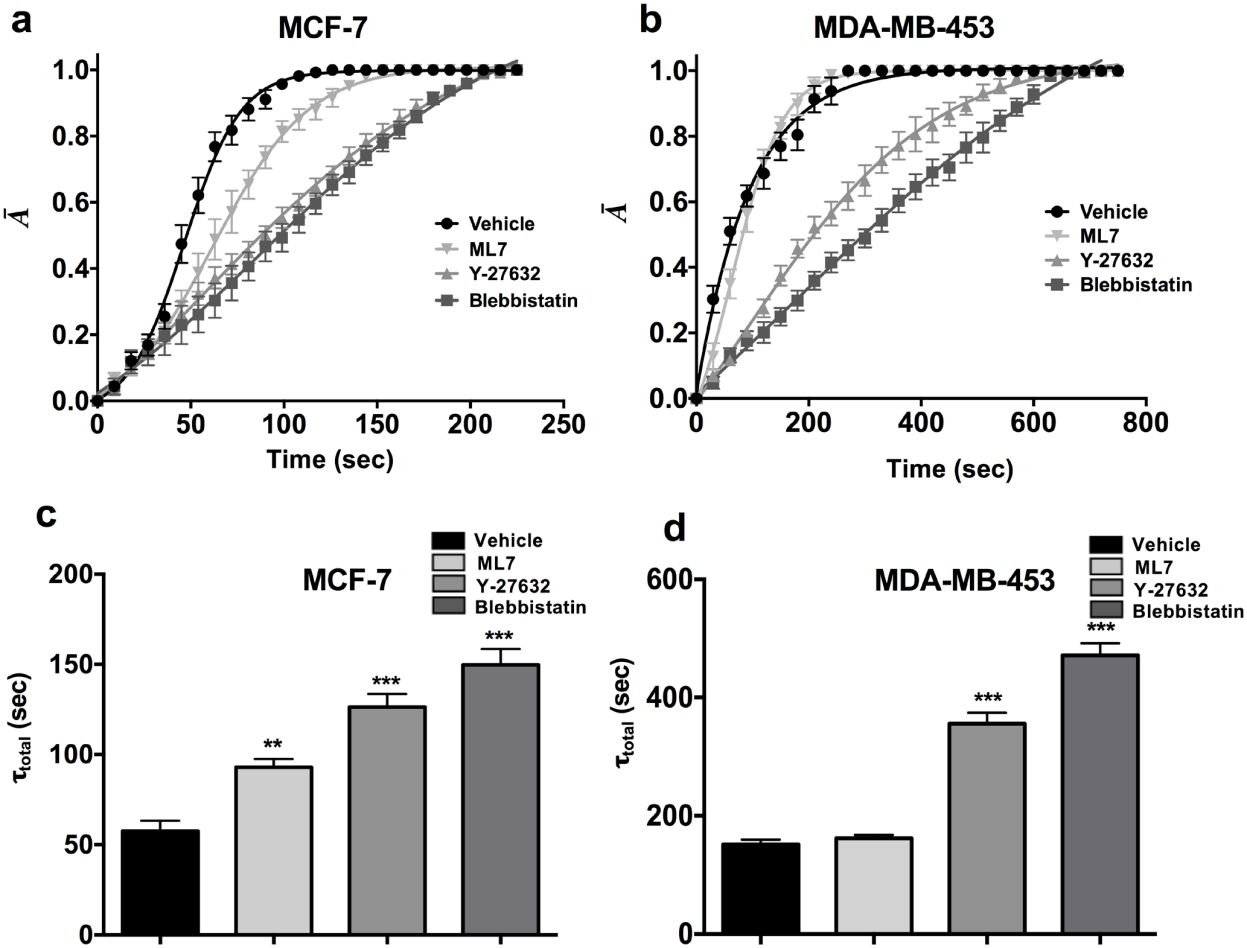
ROCK pathway is essential for maintaining contractility in parental cells **a-b**. The parental cells of MCF-7 and MDA-MB-453 treated with Blebbistatin and Y-27632 showed highly significant ablation in the contractile dynamics in both the cell lines, while ML7 treated SDC did not show a significant reduction in contractility MDA-MB-453 cells. **c-d**. Y-27632 and Blebbistatin treated cells showed a significant increase in the deadhesion time (τ_total_) as compared to the vehicle treated control in both MCF-7 and MDA-MB-453 (**, p value < 0.001) (***, p value < 0.0001). (n= 10 cells per condition). Data is represented as Mean ± SEM for line graph and Mean + SEM for bar graph.

In order to examine the effect of the pharmacological agents on the bulk tumor population, parental cells of MCF-7 and MDA-MB-453 cell lines were treated with ML7, Y-27632 and Blebbistatin (Figure 3a-3d). Similar to CSCs, Y-27632 and Blebbistatin treated parental cells showed almost 2-3 fold increase in the de-adhesion time when compared to the vehicle treated sample (Figure 3c-3d; p value <0.0001). Overall, these results suggest that the ROCK pathway regulates contractility of CSCs as well as tumor bulk population.

### Examining the cortical stiffness of CSCs by atomic force microscopy (AFM)

**Figure 4:**
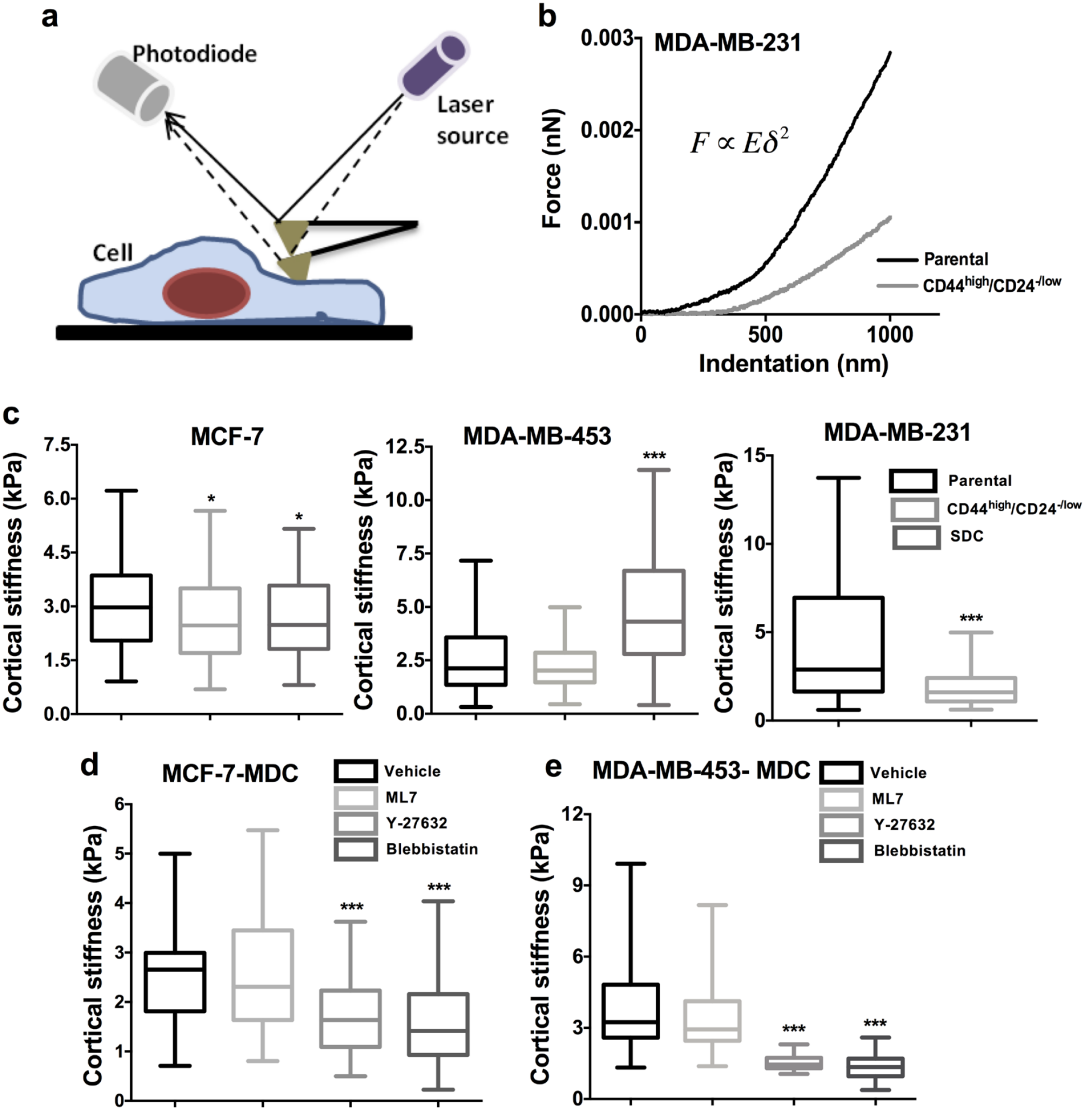
CSCs show differential cortical stiffness compared to parental cells **a**. Schematic of indentation of cell by AFM probe to examine cell stiffness. **b**. Representative force-indentation curves generated by the indentation of parental and CSCs of MDA-MB-231. **c**. Data represents the cortical stiffness of CD44^high/^CD24^−/low^ cells (MCF-7 and MDA-MB-231, and MDA-MB-453) and SDCs (MCF-7 and MDA-MB-453) compared to the parental population (n = 100 cells per condition). **d-e**. SDCs from MCF-7, and MDA-MB-453 were treated with contractility inhibiting drugs ML7, Y-27632 and Blebbistatin and cortical stiffness was quantified using AFM (*, p value < 0.05) (**, p value < 0.001) (***, p value < 0.0001) (n = 50 cells per condition). Representation: Min to max box plot with median.

Given the close association between cell stiffness and invasiveness, we next probed cortical stiffness of CSCs by atomic force microscopy (AFM) (Figure 4a). Experimental force-indentation curves were fit with Hertz model to obtain estimates of the elastic modulus of the cells (Figure 4b). CSCs obtained from MCF-7 and MDA-MB-231 using both methods (sorted CD44^high^/CD24^−/low^ and SDCs) were significantly softer than parental cells (p < 0.05). Intriguingly, MDA-MB-453 CSCs obtained from spheroids (SDCs) exhibited higher cortical stiffness (p < 0.05) and sorted CD44^high^/CD24^−/low^ CSCs of MDA-MB-453 showed no significant difference in cortical stiffness (Figure 4c).

Next, AFM was performed with CSCs (SDCs) of MDA-MB-453 and MCF-7 cell lines upon treatment with the inhibitors to probe the effects of the MLCK and ROCK pathways on cell stiffness. Y-27632 and Blebbistatin treatment reduced cell stiffness by nearly 50% in SDCs of MCF-7 and MDA-MB-453, indicating the roles of ROCK pathway in maintaining cell stiffness in both the cell lines (Figure 4d-4e).

### CSCs possess increased ECM remodeling potential

**Figure 5:**
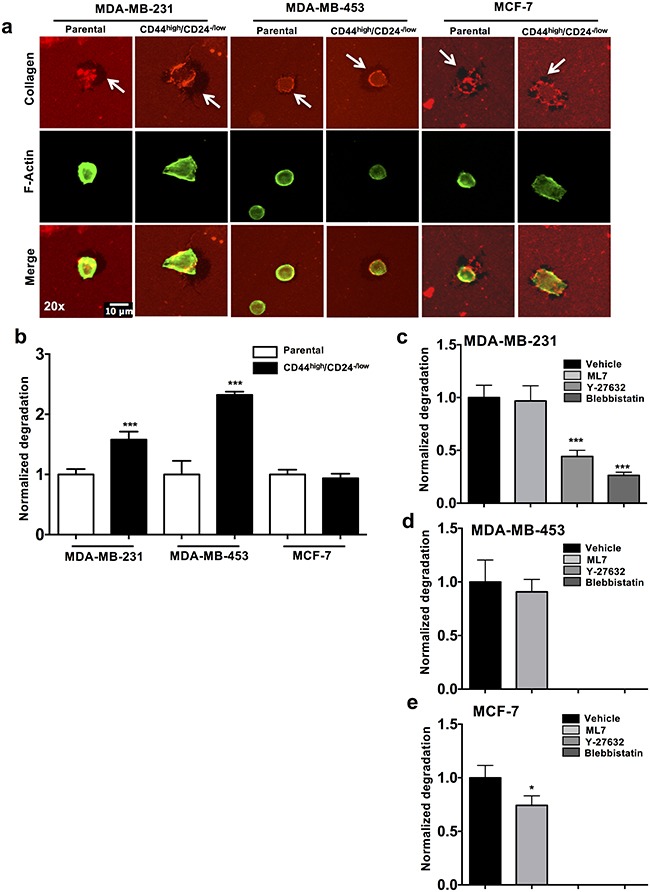
Roles of ROCK pathway in CSCs and parental cells in ECM degrading ability **a**. Representative images of collagen (red), F-actin (green) and merged staining from three cancer cells lines are depicted. Arrows indicate the area with degraded collagen. **b**. Normalized collagen degradation of CD44^high/^CD24^−/low^ CSCs was quantified keeping parental cell degradation as reference (n=3). **c-e**. Normalized collagen degradation of parental cells (MDA-MB-231, MDA-MB-453 and MCF-7) was quantified upon treatment with control (vehicle), ML7, Y-27632 and Blebbistatin. (* p value < 0.05) (*** p value < 0.0001) (n= 50 cells per condition). Representation: mean + SEM.

To examine if higher contractile dynamics of CSCs results in increase in invasion potential, we analyzed the ECM remodeling potential of CSCs and parental cells by the collagen degradation assay. CSCs (sorted CD44^high^/CD24^−/low^ cells) and parental cells were seeded on fluorescently tagged collagen-coated glass coverslips and relative degradation of the collagen compared to the cell area was quantified (Figure 5a). Significantly higher level of degradation was observed in CSCs compared to the control (parental cells) in MDA-MB-453 (2.5 fold increase, p value < 0.0001) and MDA-MB-231 (1.5 fold increase, p value < 0.0001). Cells sorted from MCF-7 did not show significant change in degradation between the CSC and parental cells (Figure 5b).

Next we examined if abrogation of cell contractility influences the collagen degradation potential of tumor cells. MCF-7, MDA-MB-453 and MDA-MB-231 cells were treated with the contractility inhibitors (ML7, Y-27632 and Blebbistatin) for 6 hours post seeding and the relative degradation of the collagen was examined. The degradation of collagen in the Y-27632 and Blebbistatin treated MDA-MB-231 cells was significantly reduced as compared to the control (p value < 0.0001) (Figure 5c). MDA-MB-453 and MCF-7 cells showed complete abrogation of degradation in Y-27632 and Blebbistatin treated cells (Figure 5d-5e). There was no significant change in the degradation capacity of the ML7 treated cells in MDA-MB-453 (Figure 5d). This indicates that abrogation of the ROCK pathway, and not MLCK pathway, reduced the ECM remodeling potential of tumor cells across all three cell lines tested.

## DISCUSSION

Given the roles of CSCs in metastasis and close association between biophysical properties of the cell and invasiveness, we speculate that CSCs possess altered cytoskeletal organization compared to non-CSCs. Our findings, for the first time, illustrate higher contractile dynamics of CSCs resulting in their increased matrix degradation potential compared with parental cells across two different types of cancers. More importantly, pharmacological inhibition of ROCK pathway abrogates the contractility and collagen degradation potential of both parental cells as well as CSCs, and may be explored as a novel strategy for targeting CSCs as well as bulk tumor population.

As a first step to compare differences in biophysical properties of CSCs and bulk population, we used two distinct methods of enriching CSCs: sorted CD44^high^/CD24^−/low^ by FACS and SDCs by spheroid assays. There have been several studies describing that CD44^high^/CD24^−/low^ population is not the exclusive biomarker for enrichment of pan-CSCs in breast cancer [4, 19–21]. Other CSCs population such as CD133+ and ALDH1+ CSCs contains the non-overlapping CSCs with CD44^high^/CD24^−/low^ population and are known to be phenotypically and functionally different [22, 23].

Cell contractility is an important factor in various cellular functions including migration, differentiation and invasion [12, 24, 25]. Seminal work by Totsukawa et al. has illustrated the role of MLCK and ROCK pathways in modulating cell contractility, with MLCK regulating peripheral stress fibers and ROCK regulating central stress fibers [17]. Our results with pharmacological inhibitors of MLCK and ROCK pathways illustrate the prominent role of ROCK pathway in cell contractility, cortical stiffness and ECM degradation of stem-like cells, and are consistent with the upregulation of Rho signaling pathway observed in various cancers [26–28]. Similarly, in fibrosarcoma, cell contractility is described to be indispensable for cell invasion in 3D matrix, with disruption of contractile machinery inhibiting ECM degradation [29]. Recently, using physiologically relevant 3D cultures, Bissell and co-workers have demonstrated that ROCK inhibition leads to reduction of cell proliferation, and decreased EGFR and integrin signaling leading to suppression of MAPK signaling [30].

Our findings in MDA-MB-231 and MCF-7 cells reveal that CSCs are softer compared to bulk population. Several recent studies have demonstrated the importance of cell stiffness on cell invasion, with cell softening associated with increased metastatic potential [31, 32]. However, the increased stiffness of CSCs isolated from MDA-MB-453 cells compared to parental cells can be attributed to the fact that they have been isolated from a melanoma cell line and are functionally different from a breast cancer cell line. Besides contractility and cell stiffness, we examined the cell shape and size of CSCs. Across all the three cell lines tested, stem like cells (sorted and SDCs) were found to be smaller in size than parental cells (Supplementary Figure 1), a factor that might directly help during invasion through dense interstitial matrices [33].

Our results demonstrate important differences in the biophysical properties of CSCs and parental cells, relevant to cancer cell invasion in *in vitro* settings. Future studies are needed to translate our findings in i*n vivo* models [34]. In addition, drugs targeting the ROCK pathway should be designed in a way that they are not easily effluxed by the drug efflux pump including ABC transporter system, which are expressed in cancer stem like cells [35–37].

## MATERIALS AND METHODS

### Cell culture

The two breast cancer cell lines (MDA-MB-231 and MCF-7) and a melanoma cell line (MDA-MB-453) were cultured in DMEM High Glucose media (Himedia, India) supplemented with 10% fetal bovine serum (FBS) and 1% penicillin-streptomycin antibiotic solution (Gibco, USA). Cells grown were incubated at 37°C in a humidified chamber with 5% CO_2_.

### Enrichment of CSCs (CD44^high^/CD24^−/low^) using FACS

Cells were harvested at 70-80% confluency and washed twice with ice cold staining buffer (1X PBS with 2% FBS). The cells were then resuspended in 50μl (per 10^6^ cells) of staining buffer and APC anti-CD44 mAb (clone: C26, 20μl/test) and FITC anti-CD24 mAb (clone: ML5, 20μl/test) (BD Biosciences, USA) were added and incubated for 30 minutes on ice in dark. Post incubation, cells were washed twice and resuspended in a final volume of 500μl of staining buffer for sorting using BD FACSAria I (Becton Dickinson, USA). The purity of sorted cells was >95%. For CD44^high^/CD24^−/low^ surface staining analysis, cell were stained, similar to as described for cell sorting, and were analyzed using BD FACSVerse system (Becton Dickinson, USA). CSCs were enriched based on surface expression of CD44 and CD24, as described previously [38]. The CD44^high^/CD24^−/low^ cells (CSCs) were sorted from MCF-7 cells and MDA-MB-231 cells. Because MDA-MB-453 cells do not express CD44 on its surface, CD24^−/low^ CSCs were sorted from these cells.

### Spheroid formation assay

MCF-7 and MDA-MB-453 cells were harvested and seeded onto non-adherent, non-tissue culture treated 6-well plates (Eppendorf, Germany) at a density of 6000 cells/well. The cells were grown in DMEM/F12 (1:1) serum free media supplemented with 10 ng/ml basic fibroblast growth factor (bFGF), 20 ng/ml epidermal growth factor (EGF), Insulin-Transferrin- Selenium (ITS, 10X) and B27 (5X) (all procured from Gibco, USA). The cells grown in these conditions grew as non-adherent, sphere like cluster of cells and were collected on the seventh day post seeding. The spheres were dissociated using 0.25% trypsin as previously described [18] and seeded for various experiments where single cells were required on collagen coated coverslips.

### RNA extraction and real time PCR

Total RNA was extracted from parental and spheroid cells from MCF-7 and MDA-MB-453 using the miRNeasy Mini kit (Qiagen, Germany) following the manufacturer's instructions. Reverse transcription was carried out using the Quantitect Reverse Transcription kit (Qiagen, Germany) using 1μg RNA. The cDNA levels were quantified by Applied Biosystems StepOne Plus (Applied Biosystems, USA) using the SYBR green assay (Quantinova SYBR green PCR mix, Qiagen Germany). Pre-designed primers (Quantitect Primer Assay) specific to the genes of interest were obtained from Qiagen, Germany. The qPCR results were analyzed using StepOne™ Software v2.3. GAPDH has been used as the endogenous control.

### ECM coated glass coverslip preparation

Glass coverslips (circular: 18mm and 12mm) were sterilized using 70% Ethanol and incubated with rat tail collagen type I (5μg/cm^2^) (Gibco, USA) overnight at 4°C. Post incubation, the coverslips were blocked with 2% pluronic (Dow, USA) for 20 minutes and rinsed twice with PBS. Cells were seeded at appropriate seeding densities on the collagen coated coverslips for various assays.

### Cell morphology assay

Cells were seeded onto 18mm collagen coated coverslips in duplicates at a seeding density of 1000 cells per well. The cells were fixed 24 hours post seeding with 4% paraformaldehyde for 20 minutes and washed twice with 1x PBS post fixing. Images of the cells were taken with Olympus 1×71 microscope. Cell area and circularity of the cells was analyzed using ImageJ software.

### Collagen degradation assay

Post blocking of the collagen (12mm coverslips), the collagen was tagged using Collagen antibody (raised in mouse) (Abcam, UK) at 1:500 dilution for 24 hours at 4°C. The coverslips were washed with 1X PBS and incubated with 1:1000 diluted Fluorescence tagged secondary antibody (Donkey anti-rabbit 555) for 2 hours at room temperature as previously described [39]. Post incubation, the coverslips were washed twice with PBS and cells were seeded at a density of 1000 cells per cm^2^ in triplicates. 6 hours post seeding, the cells were fixed with 4% paraformaldehyde and washed twice with PBS. Post washing, blocking was done using 1% BSA for 1 hour at room temperature. F-actin staining was done to visualize the cells using Alexa Fluor 488 Phalloidin antibody at a dilution of 1:500 (in PBS) for 90 minutes at room temperature. Images of the tagged collagen and actin were taken for individual cells on an Olympus 1×71 microscope at 20x magnification. The extent of degradation of the collagen was quantified by thresholding the collagen images using ImageJ software. This was normalized using the cell spread area from the F-actin staining.

### Trypsin deadhesion assay

Cells were seeded at seeding density of 1000 cells/cm^2^ on 18 mm coverslip. The experiment was performed after 24 hours of incubation. The cells were washed with warm PBS (37°C) and pre-warmed trypsin (0.25%, 500 μl) was then added to the well. Live cell images were captured every 3 seconds using the Olympus 1×71 microscope till the cells rounded up but remained attached to the substrate. For quantifying de-adhesion dynamics, the normalized change in area ($\bar{A}$) was calculated using the formula ($\bar{A}=\frac{A_{i}-A_{t}}{A_{i}-A_{f}}$), where *A_i_* represents the cell area at time t=0, *A_t_* represents the area at time t, and *A_f_* represents the area at the final time point. The experimental de-adhesion curves were fitted with the Boltzmann equation ($\bar{A}=\frac{1}{1+e^{\left(t-\tau l\right)/\tau 2}}$) to obtain the de-adhesion time constants τ_1_ and τ_2_, respectively. τ_total_ was calculated as the sum of τ_1_ and τ_2_ [7].

### Atomic force microscopy (AFM)

Cells were seeded on 18mm coverslips coated with collagen and allowed to adhere for a minimum of 4 hours. AFM measurements were performed with an Asylum MFP3DAFM (Asylum Research, CA) coupled to a Nikon TE2000E2 epifluorescence microscope. Individual cells were indented using a pyramid-tipped probe (Olympus) with a nominal spring constant of 10 pN/nm. The probe was first calibrated in air using thermal vibration method to determine the exact spring constant. The first 1μm of force-indentation curves for individual cells were fitted with the Hertzian model for a pyramidal tip to obtain estimates of cortical stiffness. At least 100 cells across three experiments were analyzed per condition.

### Treatment of cells with contractility inhibitors

To modulate the contractility of the cells in various experiments, contractility altering drugs including ML7 (Myosin Light Chain Kinase inhibitor) Y-27632 (Rho-associated protein Kinase inhibitor) and Blebbistatin (a global Non-Muscle Myosin II inhibitor) [40], were used. The drugs were used at the final concentration of 10μM for Blebbistatin and 5μM for Y-27632 and ML7. DMSO was used as the vehicle control.

### Statistical analysis

Statistical analysis for all the experiments was performed using the standard student's t-test. Statistical significance was designated based on the p value as: *: p value < 0.05, **: p value < 0.001, ***: p value < 0.0001.

## SUPPLEMENTARY MATERIALS FIGURES AND TABLES



## Abbreviations

ROCK: Rho associated protein kinase
CSC: Cancer stem like cells
AFM: Atomic force microscopy
TNBC: Triple negative breast cancer
ECM: Extra cellular matrix
MMP: Matrix metalloproteinase
MLCK: Myosin light chain kinase
MLC: Myosin light chain
FBS: Fetal bovine serum
DMEM: Dulbecco's modified Eagle's medium
APC: Allophycocyanin
FITC: Fluorescein isothiocyanate
FACS: Fluorescence activated cell sorting
FGF: Fibroblast growth factor
EGF: Epidermal growth factor
ITS: Insulin-Transferrin-Selenium
BSA: Bovine serum albumin
DMSO: Dimethyl sulphoxide
RT-PCR: Reverse transcription polymerase chain reaction
SDC: Spheroid derived single cells
ALDH: Aldehyde dehydrogenase
