# Supplementary material for: Blockade of Rho-associated protein kinase (ROCK) inhibits the contractility and invasion potential of cancer stem like cells
Source: Oncotarget. 2017 Feb 10;8(13):21418–28. doi: 10.18632/oncotarget.15248 (PMC5400594; doi:10.18632/oncotarget.15248)
Supplement: Supplementary file 1 [file oncotarget-08-21418-s001.pdf]

# Blockade of Rho-associated protein kinase (ROCK) inhibits the contractility and invasion potential of cancer stem like cells

## SUPPLEMENTARY FIGURE

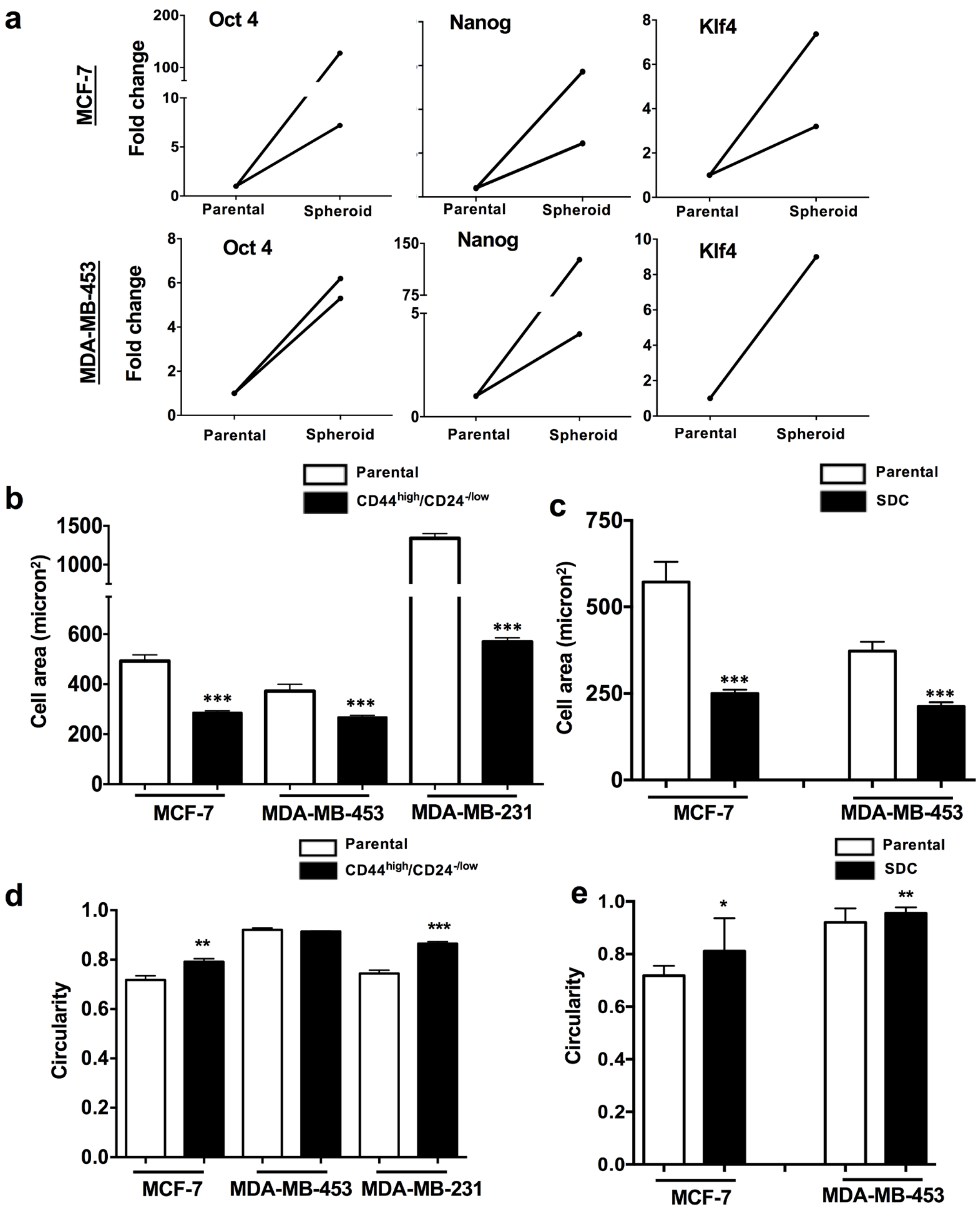


**Supplementary Figure 1: a.** Spheroids show increased expression of stemness markers (mRNA levels) such as Oct4, Nanog and Klf4 when compared to the control (parental population) as determined by Real time RT-PCR. **b-c.** Quantification of cell area of CSCs (CD44high/CD24-/low cells and SDCs) compared with control cells (parental) from all three cell lines. All three cell lines (MCF-7, MDA-MB-231, and MDA-MB-453) showed significant decrease in cell area in both CD44high/CD24-/low and SDC when compared to the parental cells. (***, $p < 0.05$) (n=3). **d-e.** Circularity of CSCs enriched using both methods (FACS sorted and spheroids) from three different cell lines (MCF-7, MDA-MB-231, and MDA-MB-453) was found to be significantly higher when compared to the parental population. (*, p value < 0.05) (**, p value < 0.001) (***, p value < 0.0001) (n=3).
